# GPIHBP1 Autoantibody‐Related Hypertriglyceridemia in Children: A Report of Two Cases and a Review of Pediatric Cases From the Literature

**DOI:** 10.1002/mgg3.70228

**Published:** 2026-05-06

**Authors:** Rai‐Hseng Hsu, Wuh‐Liang Hwu, Feng‐Jung Yang, Ni‐Chung Lee, Yin‐Hsiu Chien

**Affiliations:** ^1^ Department of Medical Genetics National Taiwan University Hospital Taipei Taiwan; ^2^ Department of Pediatrics National Taiwan University Hospital Taipei Taiwan; ^3^ Department of Pediatrics National Taiwan University College of Medicine Taipei Taiwan; ^4^ Center for Precision Medicine, China Medical University Hospital Taichung Taiwan; ^5^ Division of Nephrology, Department of Internal Medicine National Taiwan University Hospital Taipei Taiwan; ^6^ Department of Internal Medicine National Taiwan University College of Medicine Taipei Taiwan

**Keywords:** autoantibody, children, glycosylphosphatidylinositol‐anchored high‐density lipoprotein‐binding protein 1 (GPIHBP1), hypertriglyceridemia

## Abstract

**Background:**

Severe hypertriglyceridemia (HTG) is rare in children and is often caused by monogenic disorders. However, autoimmune mechanisms, particularly antibodies against glycosylphosphatidylinositol‐anchored high‐density lipoprotein‐binding protein 1 (GPIHBP1), have emerged as rare causes. Pediatric‐onset GPIHBP1 autoantibody‐related HTG remains poorly characterized.

**Methods:**

We evaluated two 3‐year‐old children with extreme HTG (triglyceride level > 3000 mg/dL). Whole‐exome sequencing was performed to investigate monogenic etiologies. Autoimmune testing included antinuclear antibodies (ANAs) and anti‐Sjögren's syndrome‐related antigen A (SSA) antibodies, as well as an enzyme‐linked immunosorbent assay (ELISA) for anti‐GPIHBP1 antibodies. A clinical response to immunosuppressive therapy was assessed. A literature review of previously reported pediatric cases was conducted.

**Results:**

No pathogenic variants were identified by whole‐exome sequencing. Both patients were positive for ANAs (1:320, speckled), and one had high‐titer anti‐SSA antibodies. The ELISA confirmed the presence of anti‐GPIHBP1 antibodies in both cases. Immunosuppressive therapy with hydroxychloroquine effectively reduced the triglyceride level in one patient, while the other required additional prednisolone. At follow‐ups (ages 9 and 10), neither patient developed overt systemic autoimmune disease. A literature review revealed 13 previously reported pediatric cases, most of which were diagnosed after the age of 11 years and were frequently associated with systemic lupus erythematosus or other autoimmune disorders.

**Conclusion:**

GPIHBP1 autoantibody‐related HTG is important and potentially treatable severe pediatric HTG, representing an early manifestation of autoimmune dysregulation that requires an accurate diagnosis and longitudinal surveillance.

## Graphical abstract

We report two young children with severe hypertriglyceridemia lacking monogenic causes. Both were ANA‐positive, with confirmed anti‐GPIHBP1 antibodies. Immunosuppressive therapy (hydroxychloroquine ± prednisolone) effectively reduced triglycerides. GPIHBP1 autoantibody‐related hypertriglyceridemia is an important pediatric cause, requiring accurate diagnosis for targeted treatment and surveillance for evolving autoimmune disease.

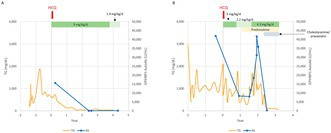

## 1 Introduction

Triglyceride (TG) levels higher than 1000 mg/dL significantly increase the risk of acute pancreatitis (AP) (Hernandez et al. 2021). Multifactorial chylomicronemia syndrome (MCS), an etiology of hypertriglyceridemia (HTG), involves both polygenic and environmental factors and is commonly associated with obesity, insulin resistance, and diabetes mellitus (Spagnuolo et al. 2024). In contrast, familial chylomicronemia syndrome (FCS) is a rare monogenic disorder that is caused by defects in genes involved in triglyceride‐rich lipoprotein metabolism, including lipoprotein lipase (*LPL*), apolipoprotein C‐II (*APOC2*), apolipoprotein A‐V (*APOA5*), and lipase maturation factor 1 (*LMF1*). Compared with patients with MCS, patients with FCS are younger and nonobese and have a higher prevalence of AP but a poorer response to current TG‐lowering strategies, including lifestyle modification and pharmacotherapy (Spagnuolo et al. 2024). The introduction of next‐generation sequencing, especially whole‐exome sequencing (WES), have greatly facilitated the diagnosis of FCS, and more genetic diseases causing HTG, including glycosylphosphatidylinositol‐anchored high‐density lipoprotein‐binding protein 1 (GPIHBP1) deficiency, have been identified (Ueda 2022; Beigneux et al. 2017). Dyslipidemia is among the manifestations of autoimmune diseases, including systemic lupus erythematosus (SLE) and rheumatoid arthritis (RA) (Szabo et al. 2017; Huang et al. 2023); however, isolated HTG without other systemic features is rare in patients with autoimmune diseases. Autoantibodies against GPIHBP1 have recently been identified as a rare cause of severe HTG and have been shown to disrupt the LPL pathway, thereby mimicking FCS in clinical presentation.

We present two cases of unrelated young children with severe HTG. WES revealed no genetic causes for the condition. Positive antinuclear antibody (ANA) tests triggered the search for autoimmune diseases, and the final diagnosis was GPIHBP1 autoantibody‐related HTG.

## 2 Materials and Methods

### 2.1 Patients

We reviewed the data of two pediatric patients diagnosed with severe HTG. We collected the initial and follow‐up clinical presentation, laboratory findings, WES results, and treatment intervention. Serial triglyceride levels and anti‐GPIHBP1 autoantibody titers were monitored during treatment and follow‐up to evaluate the biochemical responses and disease courses. The study was approved by the National Taiwan University Hospital Human Trials Ethics Committee (Ethics Committee No.: 202407171RINA).

### 2.2 Anti‐GPIHBP1 Autoantibody Assay

GPIHBP1 autoantibodies were measured by using a commercial enzyme‐linked immunosorbent assay (ELISA) kit from Immuno‐Biological Laboratories (Fujioka, Japan). Serum samples were initially diluted 1:1,000 for analysis. For samples with values above the assay range, additional dilutions (1:10,000 or 1:100,000) were performed for accurate quantification of high‐titer samples. Diluted serum samples were added to a 96‐well ELISA plate coated with purified GPIHBP1, followed by incubation with HRP‐labeled goat anti‐human IgG. After the plate was washed, color development was achieved with the 3,3′,5,5′‐tetramethylbenzidine (TMB) substrate, and the absorbance (OD) was measured at 450 nm. Autoantibody concentrations were calculated according to the kit standard curve. The assay cutoff for positivity was defined according to the manufacturer's instructions, with a reference range of 570.6–1625.6 pg/mL in human serum.

## 3 Results

**FIGURE 1:**
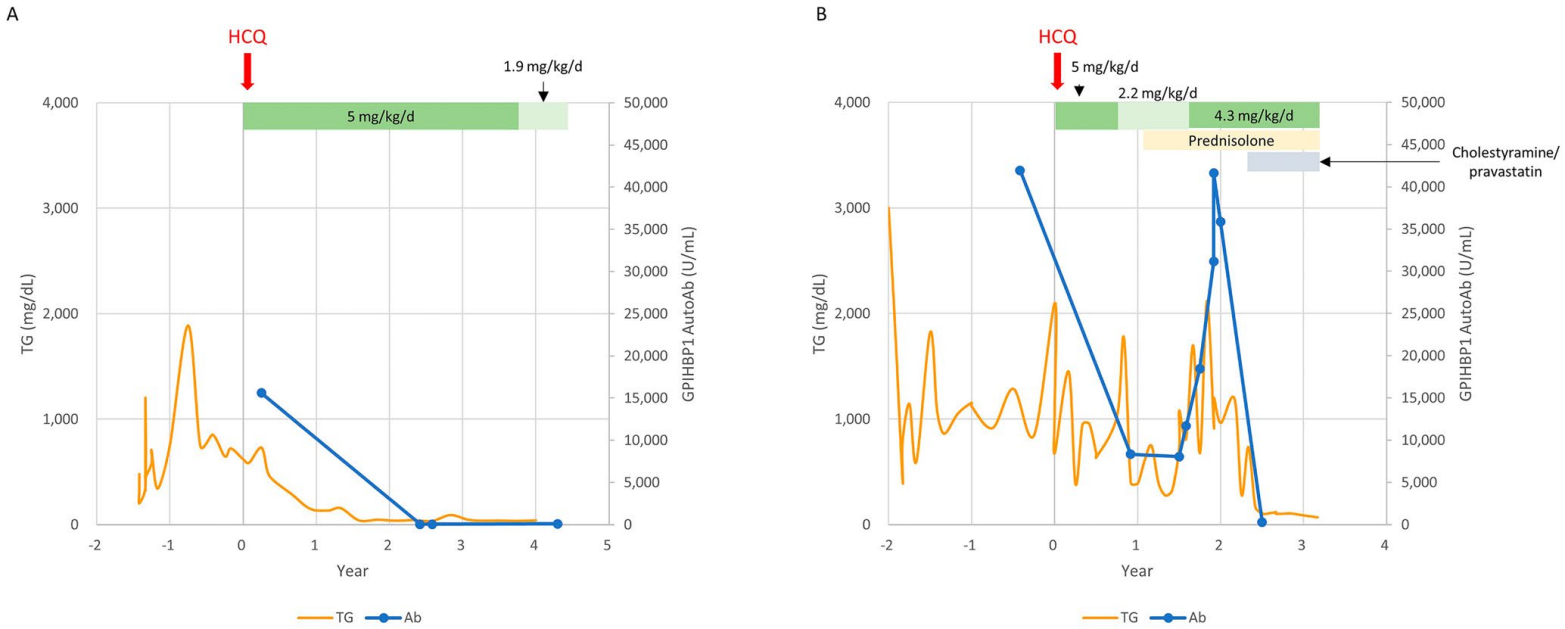
Triglyceride and GPIHBP1 autoantibody levels and medical treatment of Patient 1 (A) and 2 (B). Ab, GPIHBP1 autoantibody; HCQ, hydroxychloroquine; TG, triglyceride.

Patient 1 was a 3‐year‐old Chinese boy who presented with recurrent abdominal pain, vomiting, AP and an initial TG level of 3300 mg/dL (expected < 150 mg/dL). His body mass index (BMI) was 13.9 kg/m^2^, and all other lipid parameters were within normal limits. He was maintained on a diet with 14%–20% of total caloric intake from fat, with additional fenofibrate therapy. A WES study showed negative results for pathogenic variants. However, a persistent increase in ANA titers (1:320 speckled; normal < 1:80) was detected. Further immunological tests revealed positive anti‐SSA antibodies (≥ 240 U/mL; normal < 7 U/mL), but no specific autoimmune disease could be diagnosed. An experimental treatment with 4 mg/kg/day hydroxychloroquine (HCQ) at the age of 5 years and 11 months gradually normalized his TG level, and fenofibrate could be discontinued (Figure 1A).

Patient 2 was a Chinese girl who developed eruptive xanthomas at the age of 3 years, and her TG level was 4810 mg/dL. Her BMI was 17 kg/m^2^, and other lipid parameters showed no significant abnormalities. The WES results were negative for pathogenic variants. Her ANA test was positive (1:320 speckled), but all other markers of autoimmune diseases were negative. Treatment with HCQ (5 mg/kg/day) at the age of 6 years decreased her TG level. Her HCQ dosage was later decreased to 2.2 mg/kg/day with concomitant administration of prednisolone 2.5 mg/day because of eye discomfort and blurred vision, but her TG level rebounded. The TG level also increased during SARS‐CoV‐2 infection and vaccination. The dosage of HCQ was increased to 4.3 mg/kg/day (Figure 1B). Cholestyramine was administered, and she was subsequently switched to pravastatin as adjunctive therapy.

Given the suspicion of autoimmune HTG in both children, their LPL antibody levels were measured and found to be negative. GPIHBP1 antibody levels were subsequently tested via an ELISA. Both children were found to have high GPIHBP1 antibody levels. The GPIHBP1 antibody titer and TG level decreased in Patient 1 after HCQ treatment (Figure 1A). In Patient 2, HCQ together with prednisolone was needed to control her GPIHBP1 antibody titer and TG level (Figure 1B). Both patients achieved normalization of their triglyceride levels over several months of treatment. We summarized the characteristics of the two cases for comparison (Table 1).

**TABLE 1:** Clinical characteristics, laboratory findings, and treatment of the two patients with GPIHBP1 autoantibody‐related hypertriglyceridemia.

	Patient 1	Patient 2
Peak TG (mg/dL)	3300	4810
Clinical manifestations	AbP, vomiting, AP	Eruptive xanthoma
Age of onset	3	3
BMI	13.9	17
Other lipid abnormalities	No	No
Lipid‐lowering agents	Fenofibrates	Cholestyramine, pravastatin
Autoimmune profiles	ANA 1:320 speckled, anti‐SSA > 240 U/mL	ANA 1:320 speckled
Immunosuppressive agents	HCQ	HCQ, prednisolone

Abbreviations: AbP, abdominal pain; AP, acute pancreatitis; HCQ, hydroxychloroquine.

## 4 Discussion

FCS has a worldwide prevalence ranging from 1 in 100,000 to 1 in 1000,000 (Carrasquilla et al. 2021; Dron and Hegele 2020), but its prevalence in Taiwan is not clear. The most common gene involved in FCS among children is *LPL*, which accounts for more than 80% of cases, followed by *APOC2*, *GPIHBP1*, *APOA5*, and *LMF1* (Brahm and Hegele 2015). WES has greatly facilitated the diagnosis of monogenic HTG, and screening for genetic etiologies on the basis of a positive family history or in any child with a TG level greater than 1000 mg/dL is recommended by the National Lipid Association (Brown et al. 2020). In the absence of effective TG‐lowering agents, dietary restriction of fat (15%–20% of total caloric intake) is the mainstay of treatment in patients with FCS.

GPIHBP1 is a glycolipid‐modified anchor protein that is expressed on the surface of capillary endothelial cells, binds LPL in interstitial spaces and shuttles LPL to the capillary lumen. In patients with *GPIHBP1* gene mutations, LPL transport is interrupted, and LPL never reaches the capillary lumen, which prevents the lipolytic processing of triglyceride‐rich lipoproteins and results in HTG (Beigneux et al. 2017). Beigneux et al. first reported that anti‐GPIHBP1 antibodies could block the binding of LPL to GPIHBP1 (Beigneux et al. 2017). Autoantibodies against apolipoprotein C‐II have also been shown to cause HTG (Miyashita et al. 2020). The detection of GPIHBP1 autoantibodies is required for the diagnosis of GPIHBP1 autoantibody‐related HTG (Miyashita et al. 2020). Currently, 32 cases of GPIHBP1 autoantibody‐related HTG have been reported, 13 of which involve children (Table 2) (Miyashita et al. 2020; Hu et al. 2017; Ashraf et al. 2020; Imai et al. 2020; Kunitsu et al. 2022; Ariza et al. 2026). Immunosuppressive therapy (e.g., rituximab, steroids, and mycophenolate mofetil) is effective at reducing TG levels and preventing further episodes of pancreatitis in most adolescent and adult patients (Miyashita et al. 2020). Only half of pediatric patients receive immunosuppressants for fear of complications. This discrepancy highlights a key clinical dilemma in pediatric patients, emphasizing the need to balance the risk of immunosuppression against the complications of severe HTG. HCQ seemed to be effective in Patient 1, although HCQ has been approved only for the treatment of rheumatic autoimmune disorders (Dima et al. 2021; Nirk et al. 2020).

**TABLE 2:** Clinical data of pediatric patients with GPIHBP1 autoantibody syndrome and literature review.

No.	Sex	Age of onset (year)	Age at diagnosis (year)	Peak TG (mg/dL)	GPIHBP1 AutoAbs (U/mL)	Clinical features	Immunosuppressive agents	Response to treatment	References
1	F	NA	3	3277	29,793	Hemolytic anemia	NA	NA	Miyashita et al. (2020)
2	F	NA	11	1807	794	AP	NA	NA	Miyashita et al. (2020)
3	F	15	17	7865	2032	AP, suspect hemolytic anemia	Rituximab	Yes	Ashraf et al. (2020)
4	F	NA	15	1140	2910	SLE, SLE nephritis, anti‐phospholipid syndrome	Rituximab	Yes	Miyashita et al. (2020)
5	M	NA	11	1580	303	Hashimoto disease	NA	NA	Miyashita et al. (2020)
6	F	NA	15	2600	3250	AP	NA	NA	Miyashita et al. (2020)
7	F	NA	14	2591	3242	AP, SLE	NA	NA	Miyashita et al. (2020)
8	M	NA	13	2120	2813	NA	NA	NA	Miyashita et al. (2020)
9	F	NA	14	4440	2813	AP, suspect SLE/RA	Rituximab	Yes	Miyashita et al. (2020)
10	F	15	18	1489	NA	NA	Prednisolone	Yes	Imai et al. (2020)
11	F	13	14	2591	6012.7	AP, SLE	Prednisone, MMF	Yes	Kunitsu et al. (2022)
12	F	< 10	16	2675	> 11,124	Recurrent AbP, AP	NA	NA	Ariza et al. (2026)
13	F	0	0	9090	81	Neonatal lupus (maternal GPIHBP1 autoantibodies causing chylomicronemia)	NA	NA	Beigneux et al. (2017)
14	M	3	5	3300	15,601	Ab, vomiting, AP	HCQ	Yes	Our case
15	F	3	5	4810	41,960	Eruptive xanthoma	HCQ, prednisolone	Yes	Our case

Abbreviations: AbP, abdominal pain; AP, acute pancreatitis; HCQ, hydroxychloroquine; LPL, lipoprotein lipase; TG, triglyceride.

Among the 13 children with GPIHBP1 autoantibody‐related HTG who we identified through a literature review, four had SLE, two had hemolytic anemia, and one had Hashimoto disease (information about complications was not available for two patients) (Table 2). Our two patients are currently 8 and 7 years old and have not developed any autoimmune manifestations other than HTG. At present, whether these two patients will develop autoimmune diseases such as SLE remains uncertain. Although early treatment of autoimmune conditions increases the probability of disease remission (Doria et al. 2010; Haville and Deane 2022), the use of immunosuppressants in young children should be approached with caution.

In conclusion, we identified GPIHBP1 autoantibody‐related HTG as an important cause of HTG in children. A correct diagnosis is indispensable for these patients because a specific treatment is available, and progression to autoimmune diseases should be monitored.

## Author Contributions

Conceptualization: R.‐H.H, W.‐L.H., F.‐J.Y.; data curation: R.‐H.H., W.‐L.H., Y.‐H.C.; investigation: R.‐H.H., W.‐L.H., F.‐J.Y., N.‐C.L., Y.‐H.C.; methodology: R.‐H.H., W.‐L.H., Y.‐H.C.; visualization: R.‐H.H., W.‐L.H., Y.‐H.C.; writing – original draft: R.‐H.H., W.‐L.H.; writing – review and editing: R.‐H.H., W.‐L.H., F.‐J.Y., N.‐C.L., Y.‐H.C.

## Funding

The authors have nothing to report.

## Ethics Statement

The study was performed in accordance with the ethical standards of the Institutional Review Board of National Taiwan University Hospital (Ethics Committee No.: 202407171RINA). The need for written informed consent was waived for this chart review study.

## Consent

The need for written informed consent was waived for this chart review study.

## Conflicts of Interest

The authors declare no conflicts of interest.

## Data Availability

The data supporting this study's findings are available from the corresponding author upon reasonable request.

## References

[mgg370228-bib-0001] Ariza, M. J. , J. Rioja , V. A. Seidel , et al. 2026. “Heterogeneous Nature of Severe Hypertriglyceridemia in Childhood.” Journal of Clinical Lipidology 20, no. 3: 629–638. 41688327 10.1016/j.jacl.2025.12.022

[mgg370228-bib-0002] Ashraf, A. P. , K. Miyashita , K. Nakajima , et al. 2020. “Intermittent Chylomicronemia Caused by Intermittent GPIHBP1 Autoantibodies.” Journal of Clinical Lipidology 14, no. 2: 197–200. 32107180 10.1016/j.jacl.2020.01.012 PMC7166156

[mgg370228-bib-0003] Beigneux, A. P. , K. Miyashita , M. Ploug , et al. 2017. “Autoantibodies Against GPIHBP1 as a Cause of Hypertriglyceridemia.” New England Journal of Medicine 376, no. 17: 1647–1658. 28402248 10.1056/NEJMoa1611930 PMC5555413

[mgg370228-bib-0004] Brahm, A. J. , and R. A. Hegele . 2015. “Chylomicronaemia—Current Diagnosis and Future Therapies.” Nature Reviews. Endocrinology 11, no. 6: 352–362. 10.1038/nrendo.2015.26 25732519

[mgg370228-bib-0005] Brown, E. E. , A. C. Sturm , M. Cuchel , et al. 2020. “Genetic Testing in Dyslipidemia: A Scientific Statement From the National Lipid Association.” Journal of Clinical Lipidology 14, no. 4: 398–413. 32507592 10.1016/j.jacl.2020.04.011

[mgg370228-bib-0006] Carrasquilla, G. D. , M. R. Christiansen , and T. O. Kilpeläinen . 2021. “The Genetic Basis of Hypertriglyceridemia.” Current Atherosclerosis Reports 23, no. 8: 39. 34146174 10.1007/s11883-021-00939-y PMC8214584

[mgg370228-bib-0007] Dima, A. , C. Jurcut , and L. Arnaud . 2021. “Hydroxychloroquine in Systemic and Autoimmune Diseases: Where Are We Now?” Joint Bone Spine 88, no. 3: 105143. 33515791 10.1016/j.jbspin.2021.105143

[mgg370228-bib-0008] Doria, A. , M. Zen , M. Canova , et al. 2010. “SLE Diagnosis and Treatment: When Early Is Early.” Autoimmunity Reviews 10, no. 1: 55–60. 20813207 10.1016/j.autrev.2010.08.014

[mgg370228-bib-0009] Dron, J. S. , and R. A. Hegele . 2020. “Genetics of Hypertriglyceridemia.” Frontiers in Endocrinology 11: 455. 32793115 10.3389/fendo.2020.00455 PMC7393009

[mgg370228-bib-0010] Haville, S. , and K. D. Deane . 2022. “Pre‐RA: Can Early Diagnosis Lead to Prevention?” Best Practice & Research. Clinical Rheumatology 36, no. 1: 101737. 34991984 10.1016/j.berh.2021.101737 PMC8977282

[mgg370228-bib-0011] Hernandez, P. , N. Passi , T. Modarressi , et al. 2021. “Clinical Management of Hypertriglyceridemia in the Prevention of Cardiovascular Disease and Pancreatitis.” Current Atherosclerosis Reports 23, no. 11: 72. 34515873 10.1007/s11883-021-00962-z PMC8436578

[mgg370228-bib-0012] Hu, X. , G. M. Dallinga‐Thie , G. K. Hovingh , et al. 2017. “GPIHBP1 Autoantibodies in a Patient With Unexplained Chylomicronemia.” Journal of Clinical Lipidology 11, no. 4: 964–971. 28666713 10.1016/j.jacl.2017.05.017 PMC5568906

[mgg370228-bib-0013] Huang, S. , Z. Zhang , Y. Cui , G. Yao , X. Ma , and H. Zhang . 2023. “Dyslipidemia Is Associated With Inflammation and Organ Involvement in Systemic Lupus Erythematosus.” Clinical Rheumatology 42, no. 6: 1565–1572. 36790644 10.1007/s10067-023-06539-2 PMC10203001

[mgg370228-bib-0014] Imai, M. , H. Yamamoto , T. Hashimoto , H. Koyama , and S. Kihara . 2020. “Acquired Marked Hypertriglyceridemia With Anti‐GPIHBP1 Antibodies.” Pediatrics International 62, no. 5: 651–653. 32463576 10.1111/ped.14154

[mgg370228-bib-0015] Kunitsu, T. , M. Harada‐Shiba , T. Sato , et al. 2022. “Development of Hypertriglyceridemia due to GPIHBP1 Autoantibodies Prior to Clinical Diagnosis of Systemic Lupus Erythematosus in a 14‐Year‐Old Girl.” Allergology International 71, no. 4: 555–557. 35662538 10.1016/j.alit.2022.05.001

[mgg370228-bib-0016] Miyashita, K. , J. Lutz , L. C. Hudgins , et al. 2020. “Chylomicronemia From GPIHBP1 Autoantibodies.” Journal of Lipid Research 61, no. 11: 1365–1376. 32948662 10.1194/jlr.R120001116 PMC7604722

[mgg370228-bib-0017] Nirk, E. L. , F. Reggiori , and M. Mauthe . 2020. “Hydroxychloroquine in Rheumatic Autoimmune Disorders and Beyond.” EMBO Molecular Medicine 12, no. 8: e12476. 32715647 10.15252/emmm.202012476 PMC7411564

[mgg370228-bib-0018] Spagnuolo, C. M. , J. Wang , M. I. AD , B. A. Kennedy , and R. A. Hegele . 2024. “Comparison of Patients With Familial Chylomicronemia Syndrome and Multifactorial Chylomicronemia Syndrome.” Journal of Clinical Endocrinology & Metabolism 48: S14. 10.1210/clinem/dgae613 PMC11913094 39238074

[mgg370228-bib-0019] Szabo, M. Z. , P. Szodoray , and E. Kiss . 2017. “Dyslipidemia in Systemic Lupus Erythematosus.” Immunologic Research 65, no. 2: 543–550. 28168401 10.1007/s12026-016-8892-9

[mgg370228-bib-0020] Ueda, M. 2022. “Familial Chylomicronemia Syndrome: Importance of Diagnostic Vigilance.” Translational Pediatrics 11, no. 10: 1588–1594. 36345451 10.21037/tp-22-488 PMC9636463

